# Missed opportunities on retinopathy of prematurity: the urgency of doing more

**DOI:** 10.5935/0004-2749.20200075

**Published:** 2020

**Authors:** Martin A. Zimmermann-Paiz, Nancy C. Quezada-del Cid, Verónica Burgos-Elías, Ana M. Ordoñez-Rivas, Silvia Aceituno, Ana L. Asturias

**Affiliations:** 1 Unidad de Oftalmología Pediátrica, Estrabismo y Neuro-Oftalmología “Dra. Ana María Illescas Putzeys”, Hospital de Ojos y Oídos “Dr. Rodolfo Robles V.” Instituto de Ciencias de la Visión. Benemérito Comité Pro Ciegos y Sordos de Guatemala, Guatemala C.A; 2 Clínica de Oftalmología Pediátrica y Estrabismo, Unidad Nacional de Oftalmología, Hospital Roosevelt, Guatemala C.A

In Guatemala, a developing country with a low per capita income and a high birth rate, 13%-18% of babies that are born from mothers aged between 15 and 49 years weigh less than 2.5 kg^(1)^. As a consequence, retinopathy of prematurity (ROP) is emerging as an important public health problem^(2-3)^. ROP is one of the major avoidable causes of childhood blindness worldwide. With the increased survival rate of infants with a very low birth weight, the absolute number of children with visual impairment secondary to ROP has increased^(4)^. In Latin America, the prevalence of any ROP stage ranges from 6.6% to 82%, and the prevalence of severe ROP that requires treatment varies from 1.2% to 23.8%. Unfortunately, in many countries in Latin America, routine screening for ROP is unavailable, and services for its treatment are lacking^(5)^.

To highlight the urgency for creating a formal ROP screening program in Guatemala, we reported the characteristics of a group of patients who had terminal stages of ROP but were never screened for it and consequently untreated (missed opportunities). We detected 30 patients and collected their data for 1 year at two ophthalmological reference centers in Guatemala City. All the patients were born in hospitals outside Guatemala City, where no ROP screening program is available.

Table 1 presents the characteristics of these cases. The average age at consultation was 14.38 months (range of 3-60 months), the average birth weight was 1395.36 g (range of 794-1900 g), and the average gestational age was 31.5 weeks (range of 25-36 weeks). In 10 cases, gestational age was unknowne, but mothers indicated that their babies were born between 6 and 8 months of gestation.

**Table 1:** Characteristics of patients with missed opportunities

Age at consultation (months)	Gestational age at birth (weeks)	Birth weight (grams)	Birth place	Diagnosis (both eyes)	No (%)
Petén region					
4	26	1300	Petén	ROP IV A	3(10)
4	32	1361	Petén	ROP V	
2	29	1332	Petén	ROP IV B	
North region					
3	Unknown	1500	Alta Verapaz	ROP V	3(10)
3	32	1531	Alta Verapaz	ROP V	
48	32	1020	Baja Verapaz	ROP V	
Northeast region					
36	Unknown	908	Izabal	ROP IV B	3(10)
36	Unknown	1644	Zacapa	ROP V	
24	Unknown	1814	Chiquimula	ROP V	
Central region					
8	Unknown	1531	Escuintla	ROP V	6(20)
12	29	1503	Escuintla	ROP V	
5	Unknown	1446	Escuintla	ROP V	
5	32	1361	Escuintla	ROP V	
5	35	1361	Sacatepéquez	ROP V	
9	Unknown	1304	Sacatepéquez	ROP V	
Southeast region					
60	32	1814	Santa Rosa	ROP V	6(20)
5	33	1758	Santa Rosa	ROP V	
3	31	Unknown	Santa Rosa	ROP V	
4	33	1332	Jutiapa	ROP V	
12	Unknown	794	Jalapa	ROP V	
11	30	1900	Jalapa	ROP V	
Southwest region					
4	Unknown	1361	Quetzaltenango	ROP V	8
3	30	1270	Quetzaltenango	ROP1 V A	(26.67)
3	26	1020	Quetzaltenango	ROP IV A	
3	29	1900	Totonicapán	ROP V	
5	Unknown	1106	Retal hu leu	ROP V	
24	28	1021	Retal hu leu	ROP V	
36	32	1814	Retal hu leu	ROP V	
4	Unknown	1361	Suchitepequez	ROP V	
Metropolitan region					
6	36	1304	Guatemala	ROP V	1(3.33)
TOTAL					30(100)

In 1 year, many children with ROP became blind, so ROP was probably secondary to a high incidence of severe stages without screening.

Many hospitals located in the capital city are covered by ROP screening programs, but many premature children survive in hospitals outside this area. Hospitals in rural areas face various health system challenges, such as insufficient equipment and qualified personnel for the optimal treatment of premature patients. These hospitals also lack trained ophthalmologists who can detect and treat ROP. Other problems include difficulty in accessibility, poverty, and cultural differences, such as language. For these reasons, ROP in patients is undetected in their place of birth. In this case series, the data suggested that premature children with a wide range of weight and gestational age should be evalua ted (unusual cases). The prevalence of blindness will likely increase in the future if necessary short-term measures will not be implemented. The first steps in the development of a formal ROP detection program are already being implemented in the Health Ministry by establishing a national regulation program. However, this program has several limitations, including the lack of equipment and qualified personnel for the execution of the program. Therefore, formal treatment planning must be initiated and prioritized.
